# COVID-19 Changed the Incidence and the Pattern of Pediatric Traumas: A Single-Centre Study in a Pediatric Emergency Department

**DOI:** 10.3390/ijerph18126573

**Published:** 2021-06-18

**Authors:** Laura Ruzzini, Sergio De Salvatore, Daniela Lamberti, Pierluigi Maglione, Ilaria Piergentili, Francesca Crea, Chiara Ossella, Pier Francesco Costici

**Affiliations:** 1 Department of Orthopedics, Children’s Hospital Bambino Gesù, Palidoro, 00165 Rome, Italy; Daniela.lamberti@opbg.net (D.L.); pierluigi.maglione@opbg.net (P.M.); pierfrancesco.costici@opbg.net (P.F.C.); 2 Department of Orthopaedic and Trauma Surgery, Campus Bio-Medico University, 21 Via Álvaro del Portillo, 00128 Rome, Italy; s.desalvatore@unicampus.it (S.D.S.); ilaria.piergentili94@gmail.com (I.P.); 3 Department of Emergency, Children’s Hospital Bambino Gesù, Palidoro, 00165 Rome, Italy; Francesca.crea@opbg.net (F.C.); chiara.ossella@opbg.net (C.O.)

**Keywords:** COVID 19, epidemiology, pediatric, trauma, fracture, children, lockdown

## Abstract

The first wave of COVID-19 spread worldwide from March to May 2020. Italy was one of the countries in the world where the lockdown period was most prolonged and restrictive. To date, the impact of prolonged lockdown on pediatric traumas has not fully investigated. This paper aimed to analyze, and compare to 2019, the incidence and the fracture pattern in patients admitted to our pediatric hospital during the total lockdown period. A single-center retrospective study was performed. The data were gathered from the Pediatric Emergency Department (PED) of the Bambino Gesù Children Hospital of Rome (Palidoro). This PED is the pediatric referral center for Rome and the hub for pediatric traumas of the region. Any admission diagnosis for fracture, trauma, sprains and dislocation during the lockdown period (10 March–4 May) were included. The demographic data, diagnosis, type of treatment, fracture segment, bone involvement and time interval between trauma and presentation to the PED were recorded. In 2020, a reduction of total traumas and fractures, compared to 2019 (*p* < 0.001), occurred (81%). Superior limb and inferior limb fractures decreased in 2020 compared to 2019 (*p* < 0.05). The identification of pediatric traumas and fractures trend could be useful to reorganize the PED. Epidemiological data from the previous lockdown could be helpful to prepare the healthcare system for new pandemic waves. Moreover, sharing national statistics and correlating those to other countries’ protocols, could be helpful to solve problems in case of worldwide emergency situations.

## 1. Introduction

The number of COVID cases increased progressively between January and February 2020, reaching exponential growth in spring of 2020 [1]. Therefore, the World Health Organization (WHO) declared the COVID-19 pandemic on 11 March 2020 [2]. COVID-19 caused a considerable burden on the healthcare system worldwide. Elective surgeries were postponed, trauma patients and all the subjects with severe comorbidities (cardiac disease, cancers and diabetes, among others) were delayed in screening and treatment. Various restrictions were adopted worldwide, including national forced lockdown in many countries [3]. These restraint measures resulted in decreased population mobility, with a consequent decrease in COVID incidence and mortality [4].

The total lockdown reduced the overall influx of adult and young patients to emergency departments (ED) [5]. These data were in line with previous coronavirus epidemics. In 2003 the Severe Acute Respiratory Syndrome (SARS) and, in 2014, Middle East Respiratory Syndrome (MERS), caused a reduction of the overall number of hospitalizations [6,7]. Italy was one of the countries where the ‘first wave’ of the coronavirus infection was most notable [8]. The Italian government declared total lockdown for all the country from March 10 to May 4 of 2020. The national rules adopted in Italy included social distancing and advice concerning use of public venues. In this period, the national healthcare system had to reorganize because of the emergency, converting many hospitals in COVID centers to ensure the management of all patients. Italy was one of the countries in the world where the lockdown period was most prolonged and restrictive.

Although few pediatric hospitalizations for COVID were reported [9], an overall increase in pediatric injury rate was noticed [10]. In particular, a significant reduction of pediatric hospital admission (ranging from 48% to 67% worldwide), with a consequent increase in acuity, was reported [11,12]. Total lockdown helped to limit numbers of pediatric traumas but not prevent all injuries, as many fractures occurred at home or during the house and garden activities. Increase in social isolation, financial problems and loss of support networks were considered as risk factors for pediatric traumas.

Traumatic injuries of children occurring at home are well reported in the literature, involving burns, fractures, contusions and traumatic wounds. Traumatic injuries are the primary cause of pediatric mortality and morbidity [13]. International studies reported overall changes in the pattern of pediatric trauma and injuries [13].

From a system preparedness standpoint, it is essential to perform an accurate report on pediatric traumas during the lockdown to understand better how it would impact the pediatric healthcare system. National health statistics are attractive for an international audience, as different approaches to manage emergencies are reported between countries. Moreover, sharing national statistics and correlating those with other countries’ protocols could be helpful to solve problems in the case of worldwide emergency situations.

The aim of this paper was to evaluate if and how the pediatric trauma pattern of patients admitted to a pediatric emergency department of a COVID-19 hospital during the total lockdown period has changed.

## 2. Materials and Methods

### 2.1. Study Design

A retrospective single-center observational study was performed. The data were gathered from the PED of the Bambino Gesù Children Hospital of Rome (Palidoro). Children’s Hospital Bambino Gesù is the biggest pediatric hospital of Rome and the hub for pediatric traumas of our region (Lazio). There are two hospitals with two emergency departments, one located in the center of the city and the other in the city hinterland. During this pandemic, part of our hospital in the city hinterland was converted to a pediatric COVID 2019 regional center. Any admission diagnosis for fracture, trauma, sprains and dislocation during the Italian lockdown period (10 March–4 May) were included. For each admission, the demographics, diagnosis, type of treatment (outpatient or hospitalization), fracture segment, bone involvement and time interval between trauma and presentation to the PED were recorded. Therefore, any patients who referred a fracture to the PED were reviewed to assess the mechanism of injury and its outcome. Exclusion criteria were duplication in identification number, episodes of return to PED, lack of clinic letter available for review and referrals to other specialties.

Fracture pattern was analyzed and divided by zone of injury: upper limbs, inferior limbs and vertebral fractures. Moreover, each group was analyzed, including a deeper analysis of the fractured bones. In the upper limbs group, radius, fingers, clavicula, humerus, metacarpal, forearm and ulna fractures were assessed. In the inferior limbs group, tibia, foot fingers, fibula, metatarsal and femur were analyzed. The etiology of the trauma was investigated, involving the modality of injury (e.g., practicing sport, playing with friends) and the place (indoor or outdoor). The type of treatment received (surgery, hospital discharge or admission) was recorded for each patient.

This cohort was compared to a cohort of patients with similar characteristics in the same period one year before (10 March–4 May of 2019). The study was conducted in accordance with the 1964 Declaration of Helsinki ethical standards.

### 2.2. Statistical Analysis

Continuous variables were presented as mean and standard deviation. The categorical variables were presented as frequencies and percentage. Chi-squared tests and independent t-tests were used to compare differences between groups for categorical and continuous variables, respectively. Significance was considered for *p* < 0.05.

## 3. Results

### 3.1. Patients Characteristics

**Figure 1:**
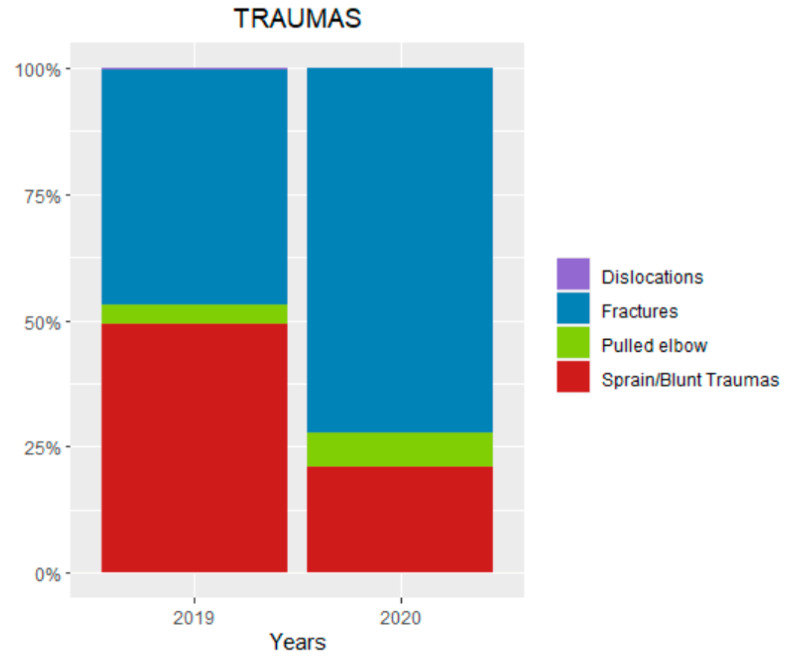
Pediatric traumas pattern during the lockdown (10 March–4 May of 2019) compared with the same period of 2019.

During the lockdown period, 173 patients were admitted to the PED with a trauma diagnosis. Of these, 125 were diagnosed for fractures, 36 for sprains or blunt traumas, and 12 for pulled elbows (Figure 1). There were 102 male patients and 71 female patients, with an average age of 8.3 years (Table 1). In the same period of 2019, there were 909 patients admitted at the PED with a diagnosis of trauma. Of these, 424 had fractures, 448 had sprains or blunt traumas, 34 had pulled elbows and five had dislocations (Figure 1). There were 512 male patients and 397 female patients, with an average age of 9.1 years (Table 1). The total amount of traumas, the mean age, the number of fractures and sprain/blunt traumas were significantly higher in 2019 than in 2020 (Table 1).

### 3.2. Time Interval between Trauma and Presentation to the Emergency Department

In 2020, the mean time between trauma and PED access was 12–24 h. Cases of malunion of forearm fractures were reported in two children, presented at the PED three weeks after trauma. By contrast, in 2019, the mean time between trauma and PED access was 3–6 h.

### 3.3. Etiology of the Trauma

**Figure 2:**
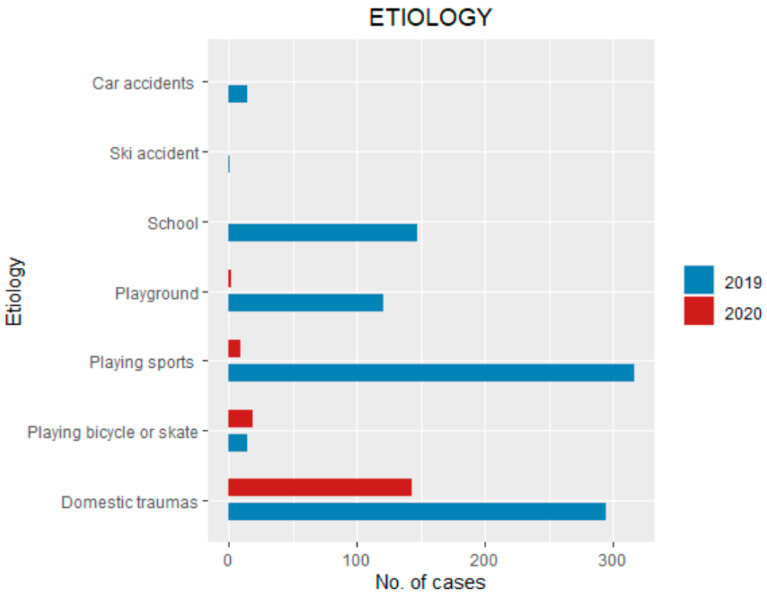
Place of accident and modality of injury of pediatric traumas during the lockdown (10 March–4 May of 2019) compared with the same period of 2019.

In 2020, 143 patients (82.6%) had domestic traumas, nine patients (10.9%) traumas on the street (riding bikes or skates), nine patients (5.2%) playing sports and two (1.1%) at the playground (Figure 2).

In 2019, 294 patients (32.3%) had domestic traumas, 316 patients (34.7%) performing sports activities, 147 patients (16.1%) traumas at school, 121 patients (13.3%) in the playground, 15 patients (1.6%) playing, bicycling or skating, one patient (0.1%) had a ski accident and15 patient s (1.6%) had car accidents (Figure 2).

### 3.4. Fracture Pattern

**Figure 3:**
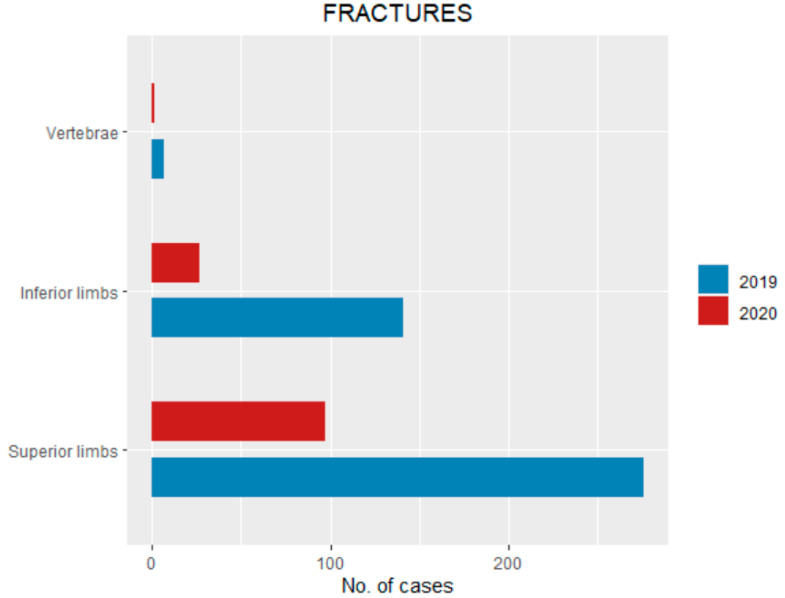
Pediatric fractures pattern (divided by groups) during the lockdown compared with the same period of 2019.

Among 125 fractures occurring in 2020, 97 (77.6%) involved superior limbs, 27 (21.6%) inferior limbs and one (0.8%) vertebrae (Figure 3). Among 424 fractures that occurred in the same period of 2019, 276 (65%) were superior limbs fractures, 141 (33.2%) were inferior limbs fractures, and 7 (1.6%) were vertebral fractures (Table 2, Figure 3).

**Figure 4:**
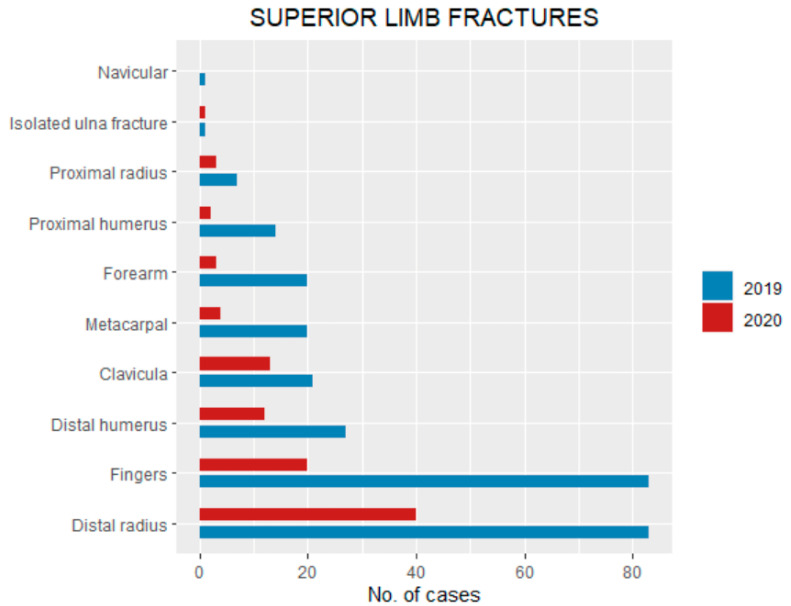
Superior limb pediatric fractures pattern (divided by bone involvement) during the lockdown compared with the same period of 2019.

Concerning superior limb fractures in 2020, the bones involved were: 40 (32% of total fractures) distal radius, 20 (16%) fingers, 13 (10.4%) clavicula, 12 (9.6%) distal humerus, four (3.2%) metacarpals, three (2.4%) forearm, three (2.4%) proximal radius, one (0.8%) proximal humerus and one (0.8%) isolated ulna fracture (Figure 4). By contrast, in 2019 superior limb bone fracture incidents were divided as follow: 83 (19.5% of total fractures) distal radius, 83 fingers (19.5%), 27 (6.3%) distal humerus, 21 (4.9%) clavicula, 20 (4.7%) forearm, 20 (4.7%) metacarpal, 10 (2.3%) proximal humerus, seven(1.6%) proximal radius, four (0.9%) isolated ulna, and one (0.2%) navicular (Table 2, Figure 4).

**Figure 5:**
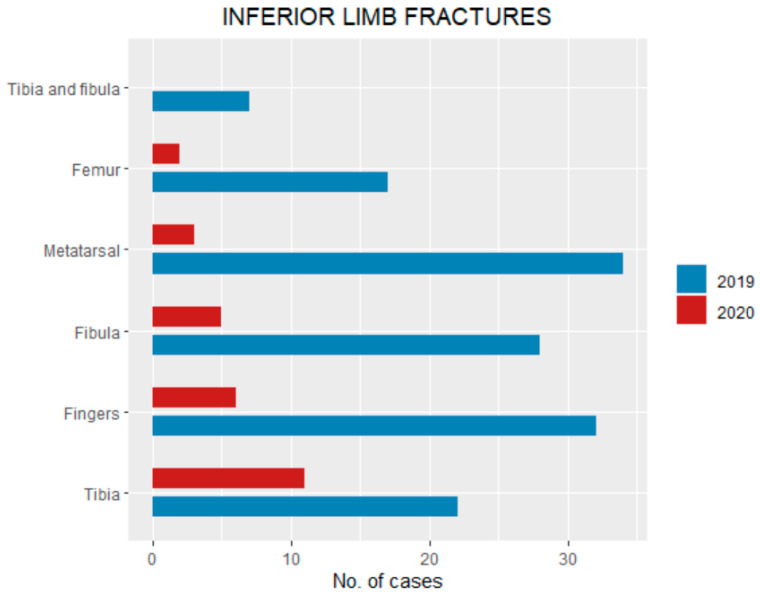
Inferior limb pediatric fractures pattern (divided by bone involvement) during the lockdown compared with the same period of 2019.

In 2020, among inferior limbs, the bones involved were: 11 tibia (8.8% of total fractures), six (4.8%) fingers, five (4%) fibula, three (2.4%) metatarsal and two (1.6%) femur (Figure 5). In 2019, the inferior limb bones involved were: 34 metatarsal (8% of total fractures), 32 (7.5%) fingers, 28 (6.6%) fibula, 22 (5.1%) tibia, 18 (4.2%) femur and seven (1.6%) tibia and fibula (Table 2, Figure 5).

Superior limbs, distal radius, inferior limbs and tibia were significantly different between 2019 and 2020 (*p* < 0.05).

### 3.5. Treatment

During the lockdown period, 10 (5.7%) patients with trauma were hospitalized, nine (5.2% of total trauma) underwent surgical treatment, and 163 (94.2%) were treated conservatively in outpatient care.

In the same period of 2019, 19 (2%) patients with trauma were hospitalized, and 16 (1.7%) underwent surgical treatment. In outpatient care, 890 (97.9%) patients were treated conservatively.

Hospitalized and Surgery were significantly different between 2019 and 2020 (*p* < 0.05).

## 4. Discussion

For the first time in the history, the entire world experienced a forced confinement due to a pandemic state. The Italian lockdown was one of the most prolonged and restrictive worldwide. The burden of COVID-19 on the healthcare system regarding the adult population has been widely assessed by many authors. However, the impact on the pediatric population has not been fully investigated. This is the first study that assesses data on COVID-19 incidence on pediatric trauma and fractures pattern in a period of lockdown. Our hospital is the reference pediatric hub of the most populous region of central and southern Italy. Therefore, the trend of certain diseases or childhood traumas recorded in the Bambino Gesù Children Hospital of Rome, allow us to reflect on the situation in most parts of Italy. In 2020, a reduction of total traumas and fractures, compared to 2019, occurred (81%). Inferior limb fractures increased in 2020 compared to 2019. A statistically significant improvement in the incidence of fractures during the lockdown, when compared with the previous year, was found. In comparison, the incidence of minor traumas was significantly reduced. A statistically significant lowering o was reported of the mean age of patients with traumas in 2020 compared with 2019.

The Covid-19 pandemic has dramatically influenced health care organization and daily life. Italy was one of the countries where this pandemic spread first at the end of February. For this reason, the Italian government decided to react with a total lockdown of the entire country from March 10 to May 4. During this period, schools, parks, playgrounds and gyms were closed, and all amateur and professional sports activities were stopped. Nonessential activities were closed, and smart working was activated where possible. These social changes had an impact on the type of pediatric traumatic injuries requiring an emergency room.

Previous papers showed that social lockdown influenced the overall number of visits to emergency departments for adult traumatic injuries, but did not influence femur fractures incidence, as these fractures occur primarily at home [14,15,16].

This may not be valid for the pediatric population. Pediatric cases of COVID contributed only between 1% to 5% of all cases worldwide [17]. It is unclear if pediatric traumas reduction was connected with the lockdown restrictions or a failure to present to the PED due to fear of visiting hospitals. The fear of hospital presentation could be act as triage selection, as reported in other specialties [18]. The delay in accessing hospital care should be prevented, and parents must be aware that the risks of delayed access for traumas could be higher than COVID-19 infection [19].

The etiology of injuries was different during the lockdown period, as most traumas occurred at home (82%) compared with 2019, during which sports accidents were the most common cause of injury (34.7%). As most fractures occurred at home, targeted measures should be considered to prevent such injuries effectively [20,21]. The superior limb was the most involved segment for fractures both in the lockdown and control groups. Lower limb and spine were less involved during the lockdown period. This could probably be explained due to the reduction of high-energy traumas (sport and motor-vehicle accidents e.g.). Changes in the overall distribution of fracture site in the superior limb were not observed except for clavicle fracture incidence that was significantly higher in 2020 (9.6% vs. 4.7%). This is probably due to the higher incidence of domestic traumas during the lockdown, as clavicle fractures usually occur after a fall from a bed or sofa [22]. Concerning the lower limb, the most involved site was the tibia, while in 2019 it was the metatarsals. There was a statistically significant higher relative incidence of fractures needing surgical management (5.2% vs. 1.7%) in 2019 compared to 2020.

The incidence of pulled elbows was higher during the lockdown (6.9% vs. 3.7%) even though the difference was not statistically significant. These data suggest adequate on-site pediatrician preparation for management of pulled elbows could have avoided access to the emergency room during the COVID pandemic period. The lower incidence of minor traumas (sprains and blunt traumas) observed during the lockdown period could also reflect abuse of PED admissions during the pre-COVID period.

The correct application of restrictive access rules to the emergency department during the COVID pandemic seems to have affected the optimization of healthcare resources. Bram et al. discussed that the COVID-19 pandemic highlighted new opportunities for simplifying patient care, such as telemedicine and management of torus distal radius fractures with splints [18]. Conservative treatment of fractures were adapted because of the significant changes that occurred during the lockdown. The use of reinforced soft casts allowed parents to manage fractures directly from home, avoiding unnecessary journeys to the hospital. Without using fixed casts, family members could, in fact, remove the soft cast themselves after being educated by physicians at the time of discharge from the PED. In addition, in some centers in the United Kingdom, a YouTube video was provided with detailed instructions for removing the soft casts [17]. An interesting solution to improve the utility of telemedicine was proposed by Tolone et al. [23]. In this paper, a questionnaire was proposed as a simple and reproducible triage procedure that might be applicable before ward admission in all hospital divisions with no emergency care, to reduce in-hospital patient care and consequent exposure of healthcare workers to contagion.

Thus, the orthopedist could follow up the patient via telephone or video chat, avoiding unnecessary travel. Of course, telemedicine has limitations for patients who require continuous monitoring to avoid complications. However, nowadays, telemedicine represents a valid, economical and useful tool in normal situations and, especially, in pandemic situations.

Sanford and colleagues [13] reported an overall change in pediatric injury and trauma pattern. Many authors reported a general decrease in blunt injuries due to the severe restriction rules adopted in each country [24]. This was probably related to the reduction of vehicle accidents and changes in childcare observation. By contrast, an increase in penetrating injuries and gun-related trauma was reported [13,25]. This data needs to be contextualized in a specific geographic area. In the United States, free access to weapons increases the risk of penetrating injuries. In particular, during a home confinement period, the accessibility to weapons is easier for children, increasing this type of injury [26,27]. In the United Kingdome, an increase of pediatric abusive head trauma was reported during the lockdown period [28]. Therefore, understanding the specific type of trauma or fracture during a confinement period could help society and healthcare systems to adopt specific prevention measures to avoid pediatric injuries.

The significant reduction in patient volumes experienced by hospitals during the 2020s may have contributed to the financial instability of many hospitals and health systems. Pelletier et al. [1] reported a resulting reduction in operating income for hospitals. Likewise, children’s hospitals also reported a decrease in financial revenue. Therefore, understanding the trend and pattern of pediatric trauma during a pandemic period may help reduce financial losses in the future.

The main limitation of this study is its retrospective single-center design. The COVID-19 impact on a single pediatric hospital does not reflect the real impact on other orthopedic hospitals. Further multicenter studies are needed to evaluate the real impact of COVID-19 on healthcare systems and assess the possible changes in patients’ morbidities and mortality. However, this is not the first study that assesses the impact of COVID-19 on the pediatric population. Rougereau et al. performed a single-center study reporting pediatric hospital admission incidence in a teaching hospital in Paris [29]. Different to our study, Rougereau and colleagues did not thoroughly investigate the specific pattern of traumas and fractures. This distinction helps organize the PED and the clinicians to face specific traumas. Baxter et al. reported the incidence of pediatric traumas in a United Kingdom hospital [17], but the sample size was smaller compared to the present study. Moreover, the lockdown period in the United Kingdome was shorter compared to Italy.

## 5. Conclusions

During the national lockdown in Italy, our pediatric hospital recorded an unprecedented decrease in admissions to the emergency department. However, patients admitted to PED during that period usually had more severe and acute injuries than in the same period of 2019, requiring more hospitalization.

We think that our findings may help elucidate national trends of pediatric traumas during this particular period. Moreover, the early identification of trends in pediatric traumas and fractures could be helpful in clinical practice by allowing reorganization of the PED and the surgical units. Orthopedic nurses and pediatricians should work together, avoiding delays in hospital presentation and consequent complications. Lastly, research results from the previous lockdown are required to prepare the healthcare system for new pandemic waves.

## Figures and Tables

**Table 1:** Demographics of pediatric traumas during lockdown compared with the 2019 group. All the data were recorded using the Pediatric Emergency Department records. Statistical significance was considered for *p* < 0.05.

	2019	2020	
Traumas	909	173	*p* < 0.001
M/F	512/397	102/71	*p* > 0.05
Age	9.1 ± 4.04 (0–18)	8.3 ± 5.8 (0–16)	*p* < 0.05
Fractures	424	125	*p* < 0.001
Sprain/Blunt Traumas	448	36	*p* < 0.001
Pulled Elbow	34	12	*p* > 0.05
Dislocations	5	0	*p* > 0.05
Hospitalized	19	10	*p* < 0.001
Outpatient	890	163	*p* > 0.05
Surgery	16	9	*p* < 0.001

**Table 2:** Fracture pattern during lockdown compared with the 2019 group. All the data were recorded using the Pediatric Emergency Department records. Statistical significance was considered for *p* < 0.05.

Fracture Involved	Bones Involved	2019	2020	
**Superior limbs**		276	97	*p* < 0.05
	Distal radius	83	40	*p* < 0.05
	Fingers	83	20	*p* > 0.05
	Clavicula	21	13	*p* > 0.05
	Distal humerus	27	12	*p* > 0.05
	Metacarpal	20	4	*p* > 0.05
	Forearm	20	3	*p* > 0.05
	Proximal radius	7	3	*p* > 0.05
	Proximal humerus	10	1	*p* > 0.05
	Isolated ulna fracture	4	1	*p* > 0.05
	Navicular	1	0	*p* > 0.05
**Inferior limbs**		141	27	*p* < 0.05
	Tibia	22	11	*p* < 0.05
	Fingers	32	6	*p* > 0.05
	Fibula	28	5	*p* > 0.05
	Metatarsal	34	3	*p* > 0.05
	Femur	18	2	*p* > 0.05
	Tibia and fibula	7	0	*p* > 0.05
**Vertebrae**		7	1	*p* > 0.05

## Data Availability

The datasets used and/or analyzed during the current study are available from the corresponding author on reasonable request.
